# Iatrogenic Cushing Syndrome With Glucocorticoid-Induced Adrenal Insufficiency Following Prolonged Topical Clobetasol Use in an Adolescent

**DOI:** 10.7759/cureus.117155

**Published:** 2026-09-29

**Authors:** Pamela Wong, Christopher Bidder, Sairan Whitaker, Krystal Wong

**Affiliations:** 1 General Medicine, Swansea Bay University Health Board, Swansea, GBR; 2 Pediatric Endocrinology, Swansea Bay University Health Board, Swansea, GBR; 3 Dermatology, Swansea Bay University Health Board, Swansea, GBR; 4 General Medicine, Cwm Taf University Health Board, Bridgend, GBR

**Keywords:** adolescent, clobetasol, glucocorticoid-induced adrenal insufficiency, iatrogenic cushing syndrome, iatrogenic hpa axis suppression, striae, topical corticosteroids (tcs)

## Abstract

Potent topical corticosteroids can be absorbed systemically and cause both iatrogenic Cushing syndrome and suppression of endogenous cortisol production. Most paediatric reports involve infants, so clinically important toxicity in adolescents may be overlooked.

A 15-year-old girl used clobetasol propionate 0.05% ointment for six months, with a reported cumulative exposure of 250 g and no documented interim clinical review. She developed Cushingoid facial changes, extensive lower-limb striae, fatigue, impaired concentration, insomnia, and low mood. Morning cortisol was <30 nmol/L and ACTH was <5 ng/L. An initial short Synacthen test showed cortisol concentrations of <30 nmol/L at baseline and 30 minutes and 38 nmol/L at 60 minutes, confirming severely impaired adrenal reserve. Clobetasol was discontinued. The patient was started on physiological hydrocortisone replacement, educated on sick-day rules, and supplied with emergency intramuscular hydrocortisone. Serial Synacthen testing showed partial recovery at 2.5 months and adequate adrenal reserve by seven months, allowing hydrocortisone to be discontinued. Her mood subsequently improved, while the striae faded gradually.

This case demonstrates a compelling association between prolonged exposure to a very potent topical corticosteroid, clobetasol, and the development of simultaneous Cushingoid features and glucocorticoid-induced adrenal insufficiency in an adolescent. Careful diagnostic review, patient counselling, planned clinical monitoring, and recognition of adrenal suppression are important medication-safety measures when potent or very potent topical corticosteroids are prescribed.

## Introduction

Cushing syndrome describes the clinical consequences of prolonged exposure to supraphysiological glucocorticoid activity. In children, the most common cause is exogenous glucocorticoid treatment, whereas endogenous Cushing syndrome is rare [1,2]. Clinical features vary with the potency, dose, and duration of exposure and may include Cushingoid facial rounding, central obesity, violaceous striae, skin thinning, proximal muscle weakness, hypertension, metabolic disturbance, and neuropsychiatric symptoms. Growth deceleration is particularly important in children and can help distinguish pathological glucocorticoid excess from uncomplicated obesity [1,2].

Although systemic corticosteroids are most frequently implicated, potent topical corticosteroids can also be absorbed sufficiently to cause significant systemic adverse effects. A historical literature review identified at least 43 cases of iatrogenic Cushing syndrome associated with very potent topical clobetasol in children and adults; most paediatric reports involved infants treated for diaper dermatitis [3]. A paediatric meta-analysis also found greater biochemical hypothalamic-pituitary-adrenal (HPA)-axis suppression with more potent topical preparations, although clinically significant adrenal insufficiency remained uncommon when lower-potency agents were used appropriately [4]. Reports of topical corticosteroid-induced Cushing syndrome in the paediatric population have predominantly involved infants and young children, which may lead to under-recognition of clinically important toxicity in adolescents.

We report an adolescent girl who developed iatrogenic Cushing syndrome with glucocorticoid-induced adrenal insufficiency (GIAI) following prolonged use of topical clobetasol propionate 0.05%. This case highlights the importance of appropriate prescribing, patient counselling, clinical review, and vigilance for systemic complications associated with very potent topical corticosteroids.

This article was presented orally at the 9th Global Virtual Conference on Pediatrics and Neonatology held on 11-12 August 2025.

## Case presentation

A 15-year-old girl presented to her general practitioner with a four-month history of a pruritic rash affecting the periumbilical region (Figure 1), hands (Figure 2) and forearm (Figure 3), following exposure to dust during home renovation. The rash was diagnosed in primary care as probable dermatitis or eczema. Initial treatment consisted of betamethasone valerate 0.025% ointment, a moderately potent corticosteroid applied twice daily for two weeks in combination with emollient therapy. On review seven weeks later, the rash did not improve, and treatment was escalated to clobetasol propionate 0.05% (Dermovate), a very potent topical corticosteroid. The rationale for escalation directly to clobetasol rather than an intermediate-potency corticosteroid was not documented in the medical record. 

**Figure 1:**
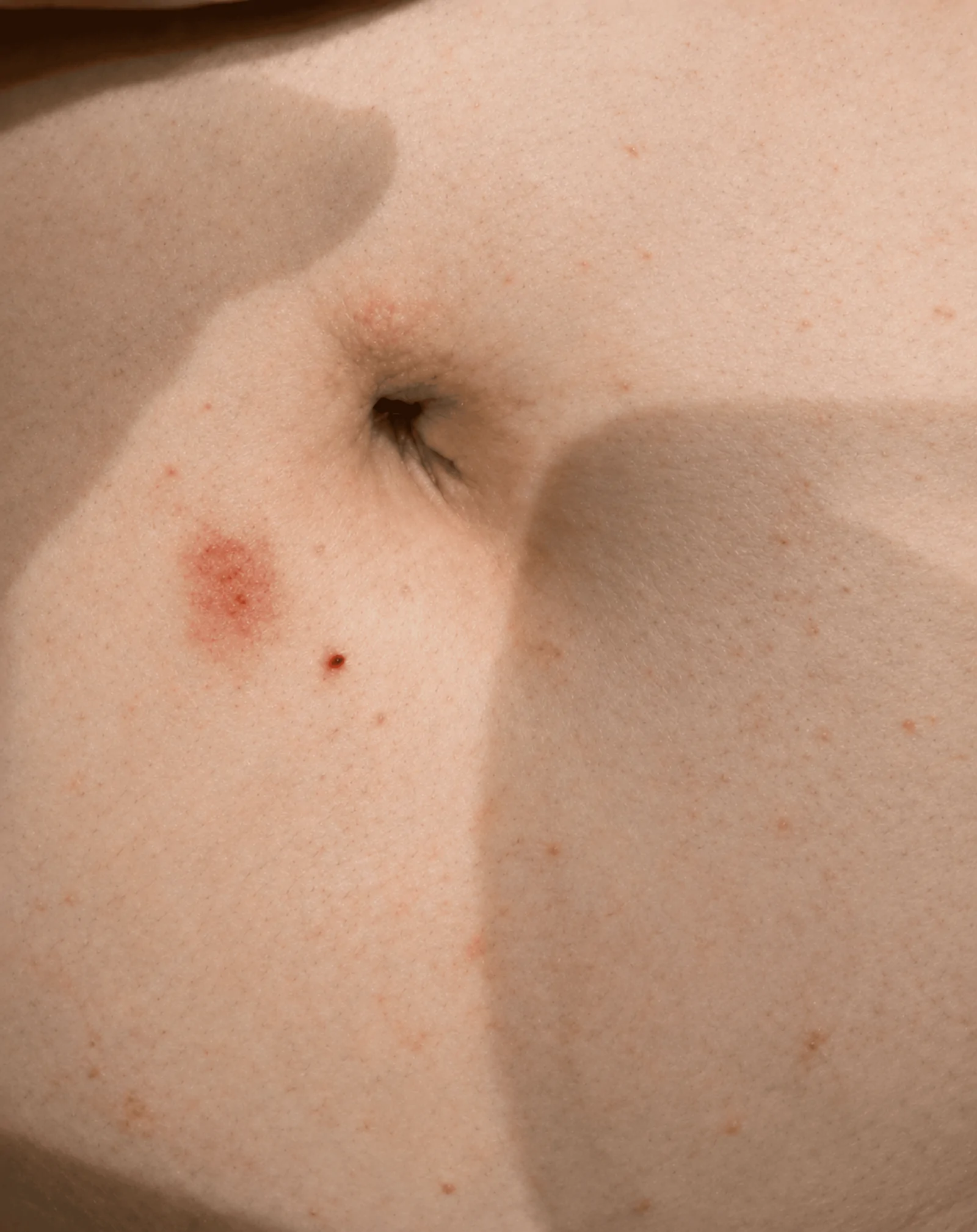
Periumbilical cutaneous eruption at initial presentation to the general practitioner following dust exposure during home renovation, before topical corticosteroid therapy.

**Figure 2:**
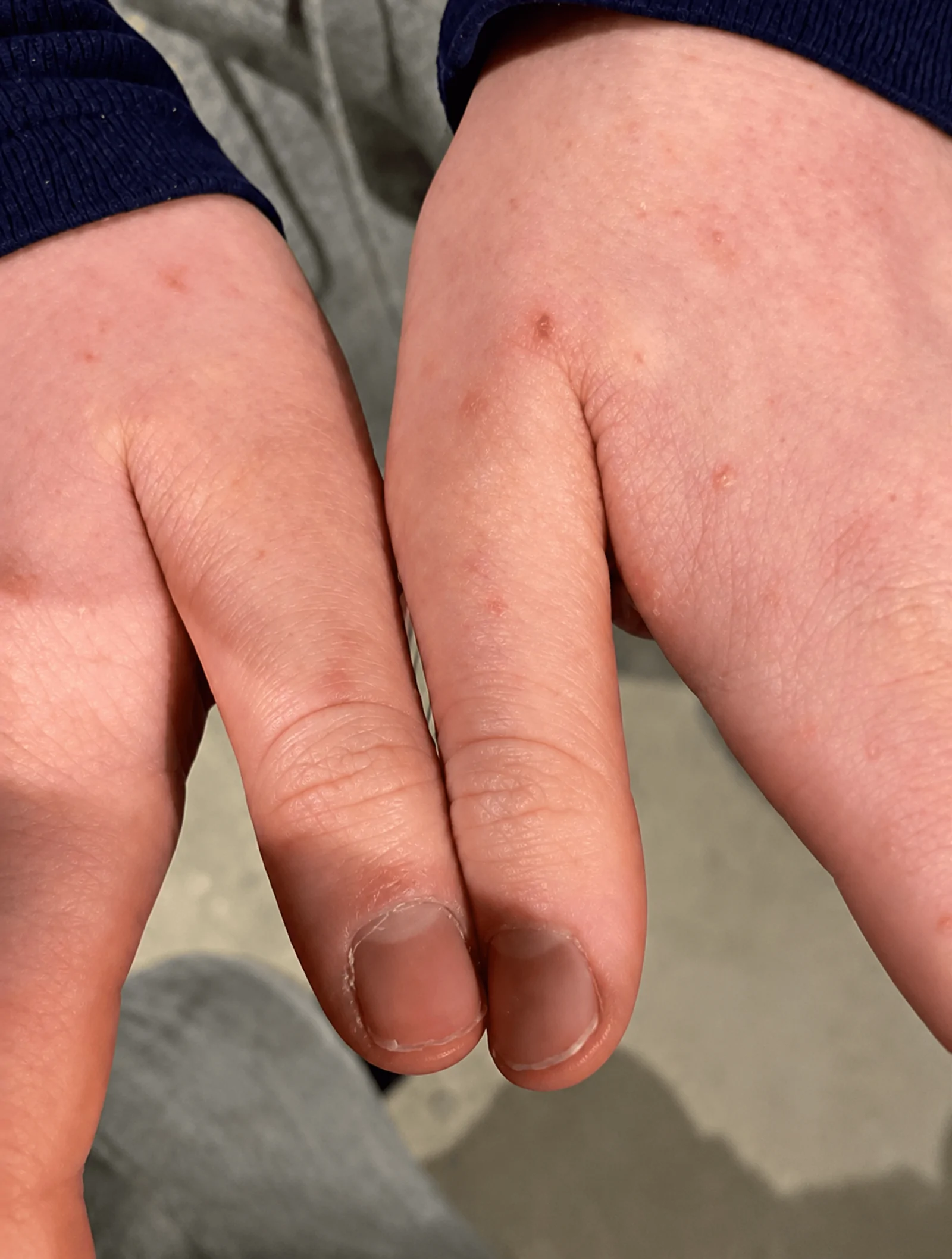
Cutaneous eruption on hands at initial presentation to the general practitioner following dust exposure during home renovation, before commencement of topical corticosteroid therapy.

**Figure 3:**
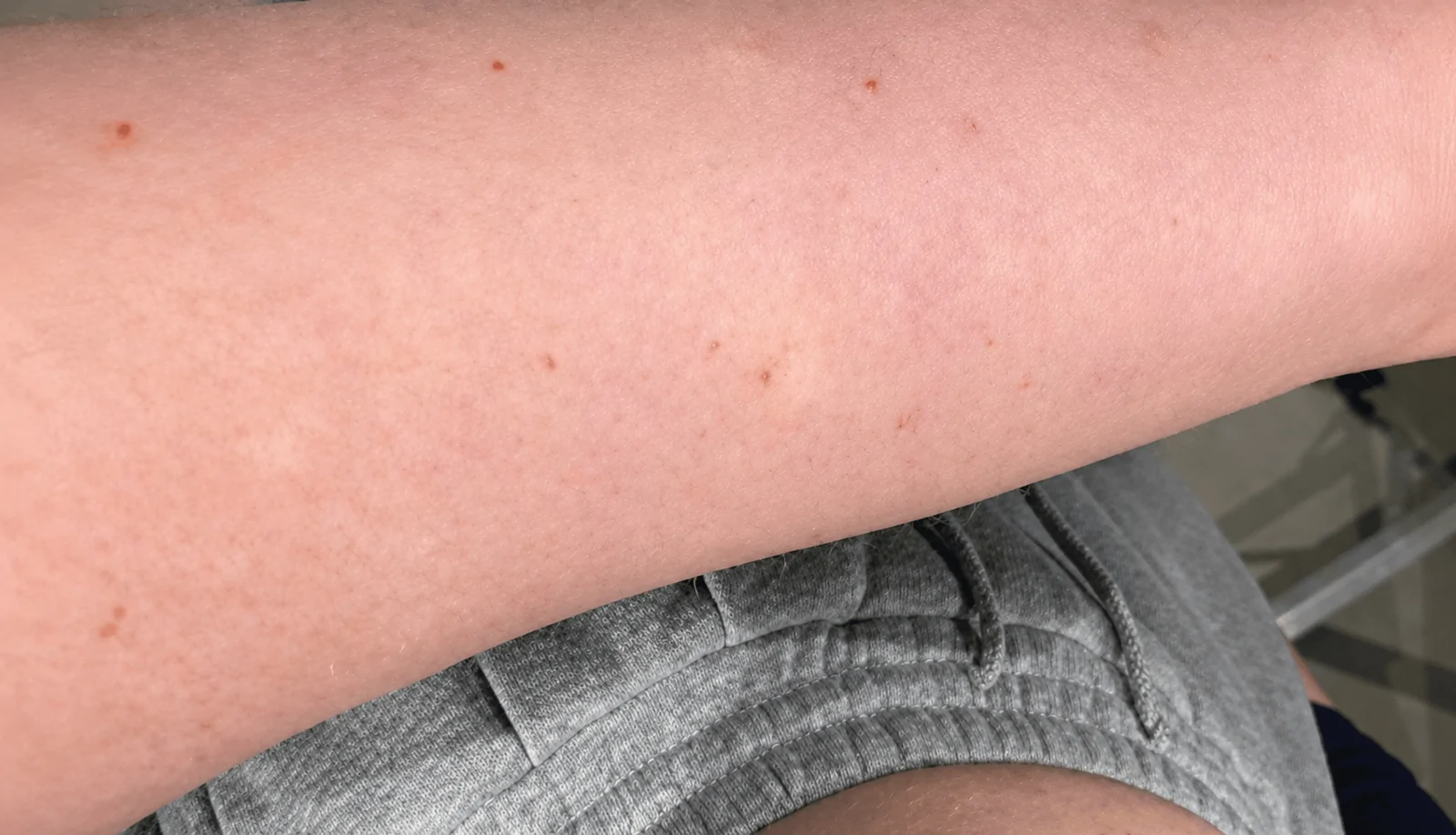
Forearm cutaneous eruption at initial presentation to the general practitioner following dust exposure during home renovation, before commencement of topical corticosteroid therapy.

Clobetasol propionate 0.05% was prescribed in 100 g tubes. General practice records documented three prescriptions without documented interim clinical review. Over six months, the patient and her parent reported using 2.5 tubes, which corresponds to an estimated cumulative exposure of 250 g of clobetasol (about 10 g/week). She reported applying small amounts to affected areas of the hands, forearms and trunk. No corticosteroid was intentionally applied to the thighs. Although the prescription instructed application of a thin layer twice daily, the patient and family recalled no counselling on fingertip-unit dosing, maximum treatment duration, cumulative quantity, adverse effects, or anatomical sites to avoid.

After six months of treatment, she developed extensive striae extending from the inner thighs to the calves (Figures 4-5). A morning serum cortisol measured in primary care was <30 nmol/L, prompting urgent referral to the Paediatric Assessment Unit (PAU). Further history identified fatigue, impaired concentration, insomnia and low mood. She also reported feeling disproportionately unwell during minor illnesses. Her parents had noticed Cushingoid facial changes described as "puffy face". Menarche had occurred three years earlier, and her menstrual cycles remained regular. Her medical history included migraine treated intermittently with paracetamol, ibuprofen and sumatriptan. She used no other regular medication and reported no oral, inhaled, intranasal or injected corticosteroid exposure. Family history included psoriasis in her mother and infantile eczema in her sister.

**Figure 4:**
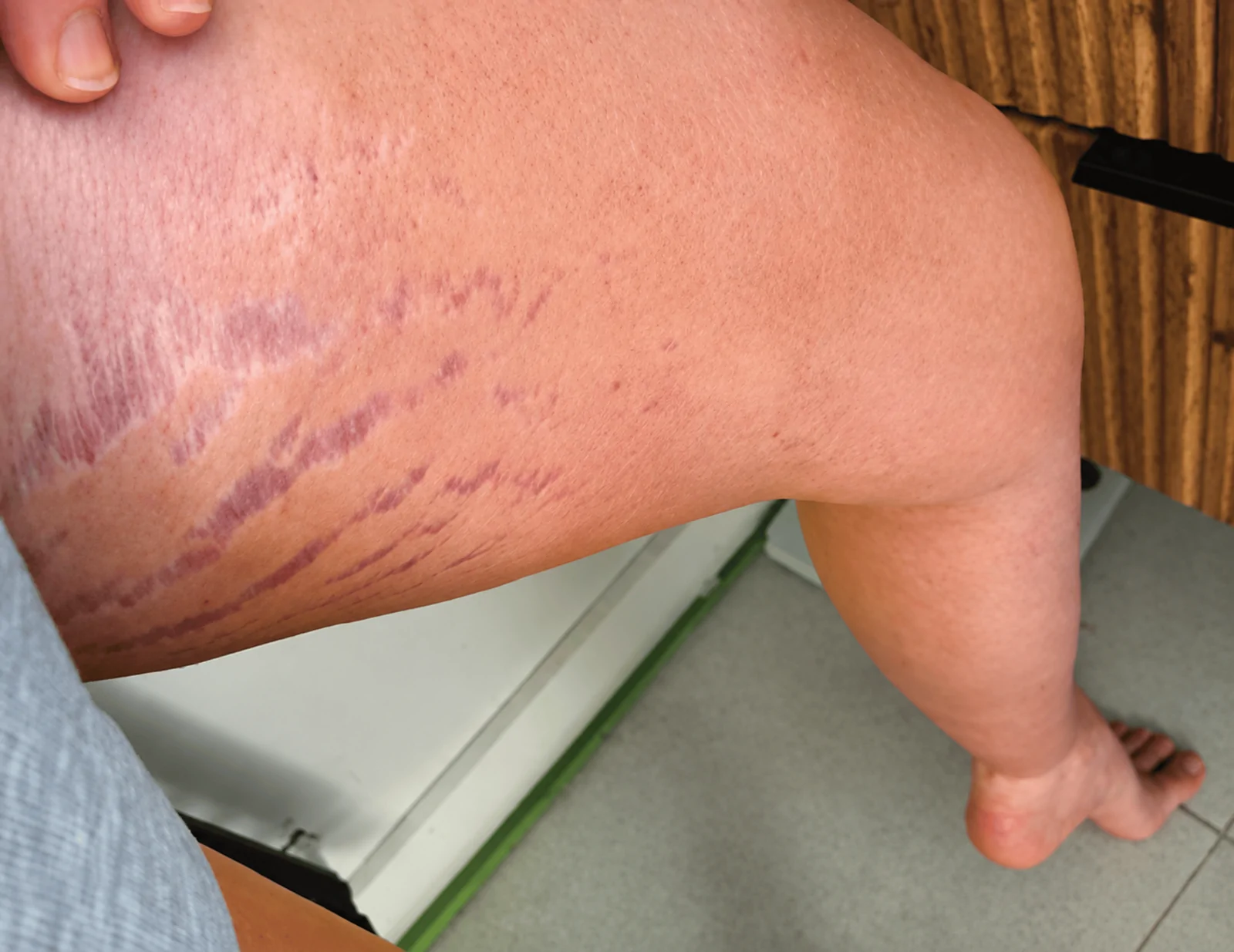
Extensive violaceous striae affecting left inner thigh after six months of topical clobetasol treatment.

**Figure 5:**
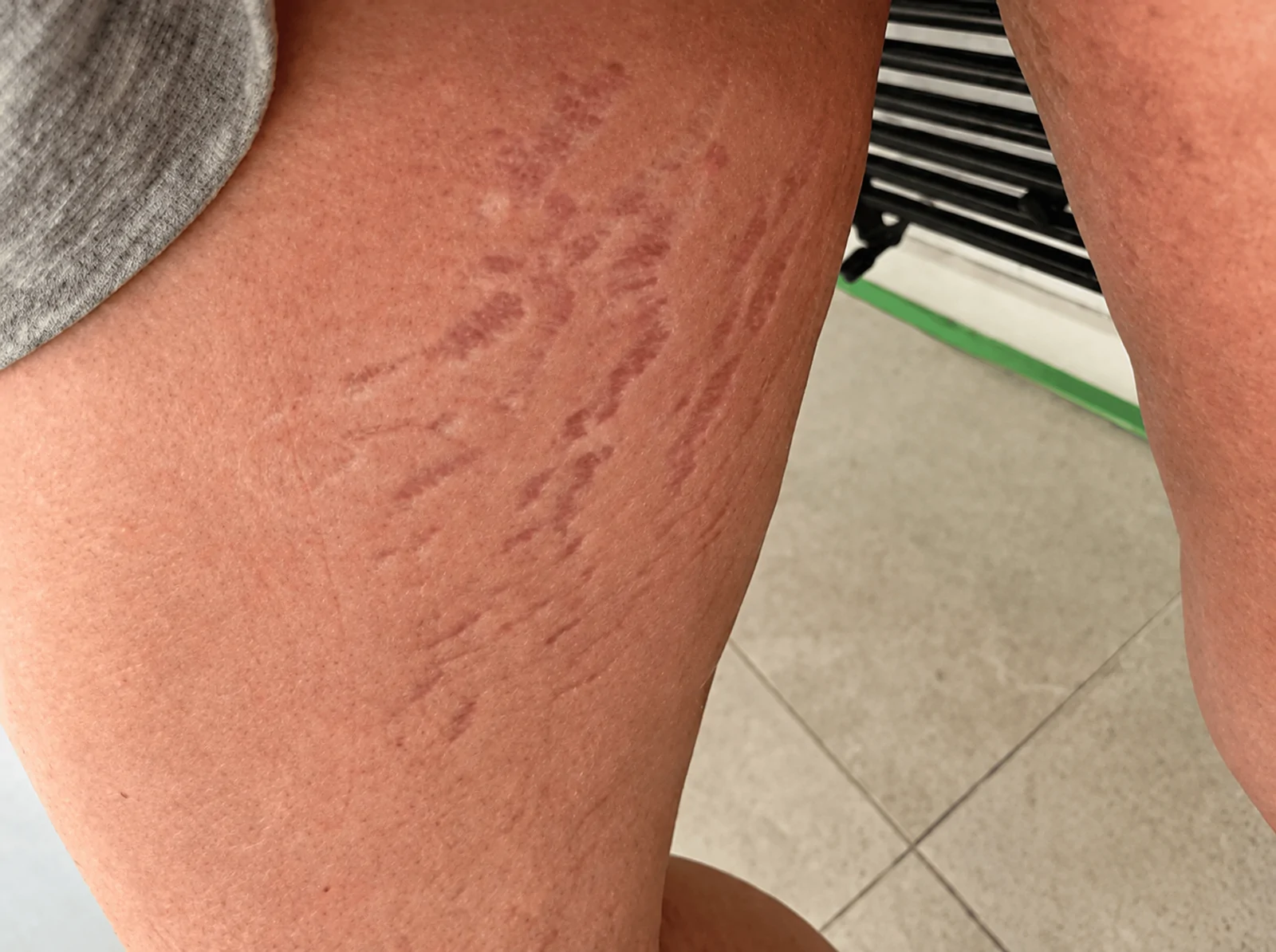
Extensive violaceous striae affecting right inner thigh after six months of topical clobetasol treatment.

She was clinically stable and well perfused. Heart rate was 65 beats/min, blood pressure 115/70 mmHg, respiratory rate 19 breaths/min, oxygen saturation 100% on room air and temperature 36.6°C. Height was 164.9 cm and weight 65.65 kg. There were no postural symptoms or hyperpigmentation. Examination demonstrated Cushingoid facial changes, residual rashes around the umbilicus, extensive lower-limb striae and delayed healing of a minor foot wound. No organomegaly or abdominal mass was detected.

Objective assessment of glucocorticoid excess was limited by the retrospective nature of the case. Growth velocity could not be reliably assessed because no contemporaneous pre-morbid height measurements were available and the patient had already reached near-adult stature, being three years post-menarche. Body Mass Index Standard Deviation Score (BMI SDS) at presentation was +1.28 (91st percentile for age and sex). Available historical records showed a 4.3 kg weight increase over the preceding 2 years and 4 months, without evidence of rapid or excessive weight gain before presentation; the patient did not report clinically significant weight gain. Blood pressure remained normal during follow-up (115/70 mmHg on presentation and 102/62 mmHg in the endocrine clinic 6 days later). HbA1c and fasting glucose were not measured.

Repeat morning cortisol remained <30 nmol/L and ACTH was suppressed at <5 ng/L. During the short Synacthen test, cortisol remained <30 nmol/L at baseline and 30 minutes and only 38 nmol/L at 60 minutes, confirming severely impaired adrenal reserve (Table 1). Sodium and potassium were normal, and adrenal antibodies were negative (Table 2). Thyroid function, insulin-like growth factor 1, gonadotropins and oestradiol were preserved; prolactin was mildly reduced at 80 mU/L (Table 3). Given the clear glucocorticoid exposure history, Cushingoid features, suppressed ACTH and cortisol and preservation of the remaining pituitary axes, structural hypothalamic-pituitary disease was considered unlikely and pituitary MRI was deferred. Subsequent recovery after clobetasol cessation further supported an acquired glucocorticoid-induced mechanism.

**Table 1:** Serial assessment of HPA-axis recovery Abbreviations: ACTH, adrenocorticotropic hormone; HPA, hypothalamic-pituitary-adrenal All short Synacthen tests used 250 micrograms of tetracosactide administered intravenously. Cortisol samples were documented clinically as baseline, 30-minute and 60-minute samples; the recorded times are shown in Table 1. Hydrocortisone was omitted on the evening before and morning of follow-up tests. All cortisol measurements were performed in the same regional laboratory using the Roche Elecsys Cortisol II immunoassay (F. Hoffmann-La Roche Ltd, Basel, Switzerland). The local laboratory used a stimulated cortisol concentration of ≥420 nmol/L as an adequate response.

Time point	ACTH (ng/L)	Short Synacthen test: cortisol (nmol/L)	Interpretation
Presentation	<5	Baseline (08:35) <30; 30 min (09:05) <30; 60 min (09:50) 38	Severely inadequate
2.5 months after clobetasol discontinued	29.1	Baseline (09:20) 212; 30 min (10:00) 260; 60 min (10:30) 295	Partial recovery; inadequate
7 months after clobetasol discontinued	19.3	Baseline (08:57) 288; 30 min (09:40) 390; 60 min (10:10) 476	Adequate recovery

**Table 2:** Initial biochemical and haematological assessment in PAU

Laboratory test	Result	Reference interval/interpretation
08:30 serum cortisol	<30 nmol/L	Morning interval 133-537 nmol/L
Sodium	139 mmol/L	135-146 mmol/L
Potassium	4.2 mmol/L	3.5-5.3 mmol/L
Urea	5.1 mmol/L	2.5-6.5 mmol/L
Creatinine	53 µmol/L	39-92 µmol/L
White cell count	10.4 × 10⁹/L	4.0-11.0 × 10⁹/L
Haemoglobin	145 g/L	115-165 g/L
Platelets	325 × 10⁹/L	150-400 × 10⁹/L
Adrenal antibodies	Negative	Negative

**Table 3:** Pituitary-function laboratory profile at presentation to PAU Abbreviations: FSH, follicle-stimulating hormone; IGF-1, insulin-like growth factor 1; LH, luteinising hormone; TSH, thyroid-stimulating hormone; free T4, free thyroxine. Reference intervals are those reported by the local laboratory. Menstrual-cycle phase was not recorded at sampling; gonadotropin and oestradiol values should therefore be interpreted in clinical context.

Laboratory test	Result	Reference interval	Interpretation
FSH	6.8 IU/L	Follicular 3.5-12.5; luteal 1.7-7.7 IU/L	Within phase-dependent range
LH	3.3 IU/L	Follicular 2.4-12.6; luteal 1.0-11.4 IU/L	Within phase-dependent range
Oestradiol	153 pmol/L	Follicular 114-332; luteal 222-854 pmol/L	Interpret with menstrual phase
IGF-1	57.0 nmol/L	15.1-72.0 nmol/L	Within range
Prolactin	80 mU/L	100-500 mU/L	Mildly reduced
TSH	2.00 mU/L	0.27-4.20 mU/L	Within range
Free T4	18.9 pmol/L	11.0-25.0 pmol/L	Within range

Clobetasol was discontinued, and Dermol 500 and Zerobase emollients were prescribed. Physiological hydrocortisone replacement was commenced at approximately 10 mg/m²/day in three divided doses (7.5 mg, 5 mg and 5 mg), with the largest dose in the morning. The patient and her family received education about sick-day dosing, symptoms of adrenal crisis and emergency intramuscular hydrocortisone. Injection kits, a steroid emergency card, medical-alert identification and a school emergency plan were provided. The endocrine team liaised with school staff and the ambulance service. Emergency intramuscular hydrocortisone was advised for clinical deterioration, persistent vomiting or diarrhoea, or inability to retain oral medication.

At one month, fatigue, significant anxiety, emotional lability and anhedonia persisted despite adherence to hydrocortisone. She had withdrawn from school activities and felt self-conscious about the appearance of the striae; psychological and school-based support were arranged. At dermatology review two months after presentation, the original rash had resolved, and eczema was considered less likely. Tacrolimus 0.1% ointment was recommended twice daily for future flares for up to three weeks, followed by twice-weekly maintenance for a three-month trial. The striae persisted and continued to affect her psychologically.

At approximately 2.5 months, hydrocortisone was withheld on the preceding evening and morning before repeat Synacthen testing. Results showed partial recovery (Table 1), so hydrocortisone and stress-dose precautions were continued. At seven months, cortisol increased from 288 nmol/L at baseline to 476 nmol/L at 60 minutes, exceeding the local assay threshold for an adequate response (Table 1). Hydrocortisone was therefore discontinued. At nine months, she had been off hydrocortisone for two months and remained clinically well. Her mother described a marked improvement in mood and behaviour, stating that “it was like a cloud had lifted” and that “she giggles again.” She was discharged from endocrine follow-up. At 13 months, the striae had faded substantially, leaving mild pink-red discolouration; the potential benefits and risks of pulsed-dye laser therapy were discussed.

## Discussion

Systemic toxicity, diagnosis and endocrine management

This case demonstrates a compelling association between prolonged use of clobetasol propionate 0.05%, a very potent topical corticosteroid, and the development of a Cushingoid phenotype with GIAI. Systemic absorption of topical corticosteroids is influenced by potency, cumulative exposure, treatment duration, frequency of application, treated surface area, anatomical site, occlusion and epidermal barrier integrity [4-6]. Treatment was initially started with betamethasone valerate 0.025%, a moderately potent corticosteroid, before escalation to clobetasol propionate 0.05% [7]. Although escalation may be appropriate when disease remains uncontrolled, very potent corticosteroids require careful monitoring because of the increased risk of local and systemic adverse effects.

General prescribing principles include confirming an appropriate indication and diagnosis; selecting corticosteroid potency and formulation according to the condition, anatomical site, lesion characteristics and patient factors; and using the lowest effective potency for an appropriate duration. Prescriptions should provide clear instructions regarding the site, frequency, duration and quantity of application. Patients should be counselled on correct application, including use of the fingertip-unit method where appropriate, and on potential adverse effects such as skin atrophy and striae. Patients receiving prolonged or repeated treatment, particularly with potent or very potent preparations, over large surface areas, under occlusion, or on thin skin, should be monitored for local and systemic adverse effects, including possible HPA-axis suppression [8].

Several of these medication-safety principles were relevant to this case. The patient received an estimated cumulative exposure of 250 g of clobetasol over six months, with repeat prescribing and no documented interim clinical review. Although the eruption was initially managed as presumed dermatitis or eczema, the diagnosis was subsequently questioned, and the rationale for escalation to a very potent corticosteroid was not documented. This highlights the importance of reconsidering the diagnosis when first-line treatment fails and considering specialist dermatological assessment when diagnostic uncertainty persists before prolonged treatment with a very potent topical corticosteroid is continued. Furthermore, there was no documented counselling regarding application quantity, treatment duration, cumulative exposure, potential adverse effects, or the need for planned follow-up. Together, these factors may have contributed to delayed recognition of systemic toxicity and reinforce the importance of careful prescribing, patient education and regular monitoring when potent or very potent topical corticosteroids are prescribed.

The biochemical combination of a low serum cortisol concentration and low or inappropriately normal ACTH suggested central HPA-axis impairment. In this patient, the diagnosis of GIAI was supported by the temporal relationship between clobetasol exposure and the development of Cushingoid facial changes and violaceous striae, together with suppressed cortisol and ACTH, severely impaired adrenal reserve on initial Synacthen testing and progressive biochemical recovery after clobetasol withdrawal. Although primary adrenal insufficiency is an important consideration when cortisol is low, it was less likely here because ACTH was suppressed, adrenal antibodies were negative, and hyperpigmentation was absent. Normal electrolytes, including potassium, were consistent with preserved mineralocorticoid function, but did not independently exclude primary adrenal insufficiency. In children, other differential diagnoses include exogenous glucocorticoid exposure, acquired hypothalamic or pituitary disease, congenital hypopituitarism and selected medication-related causes [9]. 

Structural hypothalamic-pituitary disease was also considered less likely. There had been no change in her longstanding migrainous headaches and no visual symptoms, growth failure, pubertal disturbance, or evidence of deficiencies across several other pituitary axes. The mildly reduced prolactin concentration was not considered sufficient evidence against pituitary disease; persistent or progressive pituitary abnormalities would have prompted repeat biochemical assessment and pituitary magnetic resonance imaging.

A congenital cause was improbable because there was no history of neonatal hypoglycaemia, prolonged neonatal jaundice, midline abnormalities, childhood growth failure or pubertal disturbance [9]. Medication-related causes of central adrenal insufficiency such as long-term opioids [10], ketoconazole [11] and immune checkpoint inhibitors [12], which can suppress ACTH secretion or impair adrenal steroidogenesis, were considered but no relevant exposures were identified.

The coexistence of Cushingoid features and low endogenous cortisol is a characteristic but potentially confusing consequence of exogenous glucocorticoid exposure. Exogenous glucocorticoids exert negative feedback at the hypothalamus and pituitary, suppressing corticotropin-releasing hormone and ACTH secretion. Sustained ACTH suppression subsequently reduces endogenous cortisol production and adrenal responsiveness. Patients may therefore exhibit clinical features of glucocorticoid excess while simultaneously being unable to produce an adequate endogenous cortisol response. Contemporary guidance uses the term GIAI without requiring routine distinction between secondary and tertiary adrenal insufficiency. It therefore recommends assuming concomitant GIAI in patients who develop features of exogenous Cushing syndrome [13]. 

The initial short Synacthen test demonstrated severely impaired adrenal reserve in the context of a high pre-test probability. The test assesses adrenal responsiveness to synthetic ACTH rather than directly evaluating the entire hypothalamic-pituitary axis. Consequently, results should be interpreted alongside the clinical history, concurrent ACTH concentrations and the assay-specific cortisol threshold [9,13]. Roche Elecsys Cortisol II (F. Hoffmann-La Roche Ltd, Basel, Switzerland) yields lower cortisol values than several older immunoassays, and a 2024 comparison study proposed normal-response cut-offs of 426 nmol/L at 30 minutes and 485 nmol/L at 60 minutes [14]. The regional laboratory’s validated threshold of ≥420 nmol/L was applied consistently in this case.

Contemporary guidance recommends morning serum cortisol as the initial assessment during glucocorticoid withdrawal and does not require routine dynamic testing in all patients [13]. In this case, serial short Synacthen tests provided objective evidence of HPA-axis recovery and supported the discontinuation of hydrocortisone replacement. The same tetracosactide dose, assay platform and interpretative threshold were used throughout follow-up, allowing results to be compared over time. Together with morning cortisol measurements, these tests showed progression from profound adrenal suppression at presentation to partial recovery at 2.5 months and adequate adrenal reserve at seven months.

Physiological hydrocortisone replacement was appropriate while adrenal function remained impaired. NICE recommends hydrocortisone at 8-10 mg/m²/day in three or four divided doses for children and young people over one year and under 16 years with central adrenal insufficiency [15]. A larger morning dose was used to approximate normal circadian cortisol secretion, with smaller doses later in the day providing ongoing replacement while reducing unnecessary evening glucocorticoid exposure, which may contribute to sleep disturbance and other manifestations of glucocorticoid over-replacement [15-17]. However, conventional immediate-release divided-dose hydrocortisone therapy remains an imperfect physiological replacement strategy and should be individualised according to symptoms, growth, weight and clinical features of under- or over-replacement [16,17].

Education regarding sick-day dosing, recognition of adrenal crisis and the use of emergency intramuscular hydrocortisone is essential because adrenal crisis is preventable but remains an important cause of paediatric morbidity [15,18]. The provision of emergency hydrocortisone, a steroid emergency card, school-based emergency planning and communication with relevant healthcare providers were therefore important aspects of care in this patient. Fludrocortisone is not routinely required in GIAI because aldosterone secretion is generally preserved [13].

Recovery of the HPA axis varies substantially between individuals and should be demonstrated rather than assumed. In this case, serial morning cortisol measurements and Synacthen tests showed progressive improvement in adrenal reserve, with partial recovery at 2.5 months and restoration of adequate adrenal function by seven months. These findings allowed hydrocortisone replacement to be discontinued safely and provided objective evidence linking withdrawal of clobetasol with recovery of endogenous adrenal function.

Cutaneous and psychological consequences

The cutaneous and psychological consequences were also clinically important. Glucocorticoids impair dermal collagen production and elastic-fibre homeostasis, resulting in skin thinning, impaired wound healing and formation of striae [19]. Striae generally evolve from erythematous or violaceous striae rubrae to paler and more atrophic striae albae [20].

In a study of 50 children and adolescents, striae were associated with impaired dermatology-related quality of life [20]. However, the psychological symptoms experienced by this patient are unlikely to have been attributable solely to her striae. Fatigue, impaired concentration, insomnia, anxiety, emotional lability and low mood may occur in association with both glucocorticoid excess and adrenal insufficiency, making it difficult to determine the relative contribution of endocrine dysfunction and altered appearance to her presentation. Nevertheless, the patient reported that the visibility and extent of her striae were the main reasons she did not participate in some school and social activities. The temporal improvement in mood following endocrine recovery raises the possibility that both altered appearance and systemic glucocorticoid effects contributed to her psychological symptoms.

No available intervention reliably removes mature striae. Reviews describe potential improvements with microneedling, platelet-rich plasma, radiofrequency treatment and ablative or non-ablative fractional lasers, but the evidence is limited by small sample sizes, heterogeneous treatment protocols and inconsistent outcome measures [21].

A split-body trial reported improvement in striae albae following treatment with a non-ablative fractional 1565-nm Er:glass laser. Nevertheless, procedural treatments should be presented as potentially improving appearance rather than producing complete resolution [22]. A recent review similarly concluded that treatment responses vary and that combination and energy-based therapies require more standardised evidence [23]. In this patient, spontaneous fading was evident by 13 months. This supported cautious counselling about the likely natural evolution of the striae and the uncertain additional benefits and potential risks of procedural intervention.

The principal limitation of this report is that the quantity of clobetasol applied during each treatment episode was not measured prospectively; cumulative exposure was estimated retrospectively. The original skin disorder remained diagnostically uncertain. It was therefore not possible to determine whether impairment of the skin barrier increased systemic absorption or whether the original condition required treatment with a very potent topical corticosteroid. Nevertheless, the chronology, biochemical findings, withdrawal of clobetasol and subsequent HPA-axis recovery provide a compelling association with GIAI.

This case reinforces the importance of prescribing the lowest appropriate topical corticosteroid potency, clearly specifying the quantity, site, frequency and duration of application, arranging clinical review before repeat prescribing of potent or very potent corticosteroids and assessing for GIAI when Cushingoid features develop, even when glucocorticoid exposure is topical. 

## Conclusions

This case demonstrates a compelling association between prolonged topical clobetasol exposure and a Cushingoid phenotype with GIAI in an adolescent. Although causality cannot be established from a single case, the temporal relationship, biochemical findings and recovery after clobetasol withdrawal support systemic topical corticosteroid toxicity as the most likely explanation. Clinicians should prescribe the lowest appropriate potency, provide clear application and review instructions, and assess for adrenal suppression when Cushingoid features develop. Confirmed adrenal insufficiency requires physiological hydrocortisone replacement, sick-day education and emergency planning until HPA-axis recovery, with dermatological and psychological support for persistent physical and psychosocial effects.
